# Meet the authors: Nicholas Kluge Corrêa and Camila Trindade Galvão

**DOI:** 10.1016/j.patter.2023.100854

**Published:** 2023-10-13

**Authors:** Nicholas Kluge Corrêa, Camila Trindade Galvão, James William Santos

**Affiliations:** 1 Graduate Program in Philosophy, Pontifical Catholic University of Rio Grande do Sul, Porto Alegre, Rio Grande do Sul, Brazil; 2 University of Bonn, Bonn, North Rhine-Westphalia, Germany; 3 Graduate Program in Law, Pontifical Catholic University of Rio Grande do Sul, Porto Alegre, Rio Grande do Sul, Brazil

## Abstract

Our paper, “Worldwide AI Ethics”, constitutes a meta-analysis of AI guidelines conducted by the AI Ethics Robotics Society, a non-profit organization dedicated to promoting awareness and conducting research on ethical inquiries concerning intelligent and autonomous systems. This research stems from our curiosity about comprehending the global landscape surrounding the normative discourse centered on AI.

## Main text

### What would you like to share about your background?

**Figure:**
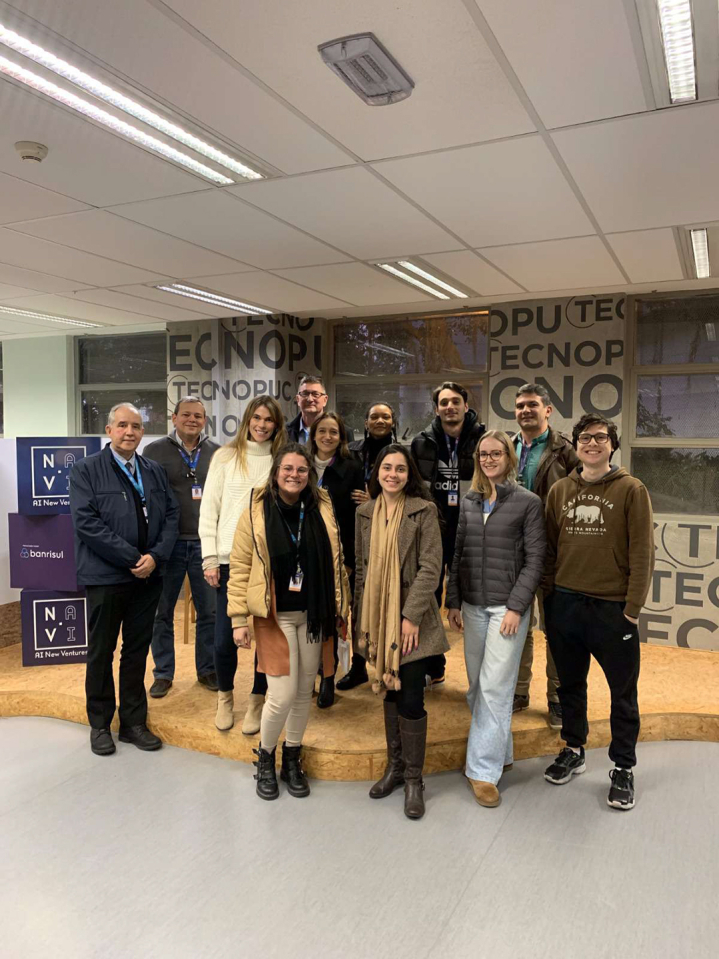
Our research was funded by the “Rede de Inteligência Artificial Ética e Segura”, a network comprised of many organizations, one of them being AIRES at PUCRS (our AIRES Chapter). In this photo, you can see our headquarters at TECNOPUC (PUCRS technology park) and part of our team. At the far left, you can see Professor Dr. Nythamar de Oliveira, coordinator of the RAIES network and one of the founders of AIRES at PUCRS. This photo was taken with the consent of the lab members photographed.

**Figure:**
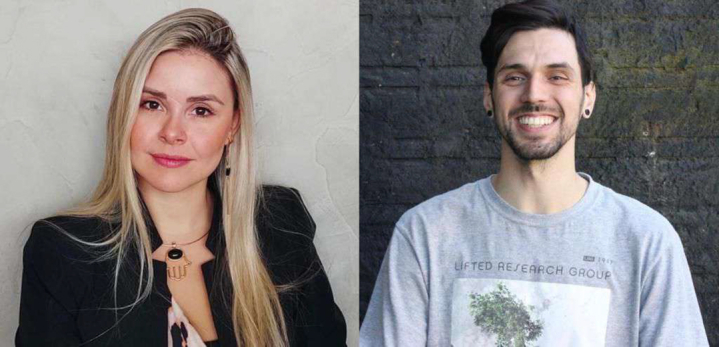
From left to right: Camila Trindade Galvão and Nicholas Kluge Corrêa

**Nicholas Kluge Corrêa:** I possess an extensive interdisciplinary background, which enables me to operate at the juncture of STEM-related domains and the humanities. Among these intersections, the realm that captivates me the most is AI ethics, along with other facets of AI research that address the challenges of normativity—that is, “What should AI do?” Engaging in the training of machine learning models and delving into their complexities has constituted a significant aspect of my academic journey since my master’s, and it remains an endeavor that I immensely relish.

**Camila Trindade Galvão:** My professional experience with technology started right after I graduated in 2014, when I worked as a consultant and advisor for technology companies, which required me to have a deeper knowledge of how artificial intelligence works in order to understand its legal aspects. In 2014, 2015, and 2016, I also directly participated in the development of part of the drafting and institutional relations with the National Congress and the executive branch for the approval of the legal framework for science and technology, which resulted in Law 13.243/2016.

### What motivated you to become a researcher? Is there anyone or anything that helped guide you on your path?

**NKC:** I chose to pursue a career as a researcher and academic primarily due to the influence of my grandfather, Vinicius. Despite lacking educational opportunities, I have been fortunate to learn from him. He was remarkably intelligent and a skilled builder and artisan. Developing a passion for construction early on, I continued to derive immense joy from creating applications, websites, models, datasets, and other things I thought might be cool and useful. In essence, research for me is a form of “building things”—whether it’s crafting a scholarly paper or an innovative new GitHub repository that I can share with others. I like researching with this “builder” mindset.

**CTG:** My history with research is very similar to that of Nicholas. My grandfather was a medical professor and his research impacted childhood AIDS and contributed to the pediatric medical assistance in our country. His legacy was recognized until he died, and I admire it. My husband is a doctoral student who also inspires me. I am a curious and inquisitive person. Since I was a child, I have tried to understand the world and academic research helps my curiosity.

### Which of the current trends in data science seem most interesting to you? In your opinion, what are the most pressing questions for the data science community?

**NKC:** The notion that data should be regarded as a “public good” deeply resonates with me. Abundant raw information exists, awaiting creative minds to transform it into tools that benefit others. This concept profoundly influences our research, compelling us to create something useful for those engaged in AI ethics, AI governance, or policy-oriented research. Moreover, I believe that one of the most pressing challenges facing the data science community today is the ease with which content and data can be created and generated. In my view, distinguishing between human-generated data and AI-generated data will be a pivotal theme in data science for the following years.

**CTG:** As I said, I’m a lawyer, and data science is changing the structure of our work and revealing other social concerns or new and silent ways to attack our rights. I believe a significant trend within data science involves the endeavor to more effectively incorporate its application into fields such as the humanities. Considering the utility of data science, this form of knowledge should be disseminated more extensively across all realms of science. In addition, ethical concerns need to be an essential part of data science if we want to grow data science with responsibility.

### Did you encounter any particular difficulties or challenges with regard to data, data management, or FAIR data sharing that you dealt with? How did you overcome them? Can others use the solutions you used to overcome these challenges?

**NKC:** As our research was conducted using publicly available documents, concerns regarding personal or sensitive information did not arise. Furthermore, unlike preceding works of a similar nature, we invested significant efforts into making our data, analyses, and method development as accessible and reproducible as feasibly achievable. All of our code and data are open to readers, facilitating the reproducibility of our findings and allowing for potential refutation if necessary. Consequently, individuals need not undertake the task of reading and parsing through the entirety of the documents we analyzed. Instead, all pertinent information has already been distilled, centralized, and made readily accessible, serving as a foundational resource for further enhanced research endeavors.

**CTG:** The biggest challenge in collecting data with a large team is standardizing interpretation. Each researcher has to read and classify a document. For example, to classify a text as being about privacy, you have to read one or many sentences and understand that it is about privacy, because the specific term “privacy” was not always there. Sometimes they said “we care about your personal data”, so we knew: this is privacy. But it is not obvious to everybody, especially when we are working with subjective concepts and many languages. So we need a lot of training and knowledge alignment, especially because we are an interdisciplinary team. After that, another difficulty was to translate complex issues and policy texts into simple terms, because we made a dashboard with a static vision about AI ethics. But we are happy with the result.

### What motivated you to publish a paper on AI ethics guidelines? Was there a particular element that motivated you to start or participate in this project?

**NKC:** We were driven by two primary motivations for conducting this research: firstly, we aimed to conduct a broader and more comprehensive analysis of the field; secondly, we endeavored to incorporate as many voices as possible into this analysis. Previous work predominantly centered around North American and European documents, which prompted us to actively seek and include perspectives from regions such as Asia, Latin America, Africa, and beyond, enriching the pool of concentrated normative discourse. Additionally, we aimed to enhance the accessibility and sharing of data and results. Had a dataset and a data visualization panel of this nature existed before our research, this paper might never have come to fruition. As such, we recognized it as a necessity and a valuable contribution to the field.

**CTG:** The idea of the research, the theme, was Nicholas’. We worked together in other projects and he invited me. I accepted because we didn’t know what countries are concerned with the challenges of artificial intelligence. Most of the studies were from the USA or EU; however, our country has a different social and economic reality. Our population can’t worry about data before worrying about hunger and unemployment. So, we worry about them. We have problems with racism, male chauvinism, the digital divide, and other prejudices developed countries don’t have on the same scale. So, we had to look for answers from realities similar to our own.

### When was AI Robotics Ethics Society founded? Would you introduce the society and your roles in the group?

**NKC and CTG:** Additionally, this research marked the inaugural undertaking of our AIRES chapter. The AI Robotics Ethics Society (AIRES) was established at UCLA in 2018 by Aaron Hui to advocate for the awareness and the significance of ethically sound implementation and regulation of AI. Presently, the society boasts numerous chapters, and the PUC-RS chapter stood as the pioneering international chapter of the organization (now AIRES has two Brazilian chapters). Nicholas holds the position of president for the PUC-RS chapter, being its founder, while Camila its our current vice president. AIRES at PUCRS functioned as a converging point for various AI-related researchers at our university, culminating in this paper that served as our maiden project.

### There is widespread public debate now about AI technologies, with some voices arguing for restrictions, slow-downs or even moratoriums on new AI technologies. Where do you stand personally on these issues? Are you optimistic or pessimistic about AI’s impact on society?

**NKC:** The origins of our society are closely intertwined with the growing excitement and concerns surrounding the advancement of AI. Nowadays, there are even pleas for the AI field to “HALT,” owing to the potential impacts that AI could have. However, perhaps due to my involvement in the field and my affinity for constructing machine learning (ML) models, I maintain considerable optimism regarding AI. I believe that in the long run, society will greatly benefit from these technologies. Currently, though, I comprehend the reasons why many individuals harbor apprehensions due to this rapid progress (everything is happening too fast …). However, in my perspective, we face truly existential threats, such as the climate crisis, which imperil our existence and have nothing to do with the new hottest chat model. These issues merit far more attention and hype than the mere capability of an natural language processing (NLP) model to generate human-like text that can be used for dubious reasons.

**CTG:** One of our missions (AIRES PUCRS BRAZIL), which has been confirmed by our research, is to bring the Global South into the discussion on ethics in artificial intelligence. As the open letter on our website says:

“This is not so much a claim as an empirical fact about the current state of the world. The vast majority of ethical guidelines and documents created to regulate and govern AI and its industry have their origins linked to organizations in the Global North. Virtually the entire Global South is outside the debate on the ethical principles that should regulate and direct the future technological development of our society. (…) Regions such as Latin America, sub-Saharan Africa, the Middle East and South Asia still have a very small voice in this debate. Other countries and cultures cannot be expected to define what is best for the context of these places. Places marked by deep scars from the old world, by colonial wounds and imperialist policies that will not be healed any time soon.”

Our research demonstrates^1^ and reinforces our call: for the Global South to wake up. And a plea for the Global North to be ready to listen and welcome us. If we really want AI to be “a warm light for all humanity to share,” we must not forget that we live in a plural, unequal, and diverse world. We must remember the voices that, until now, haven’t had the opportunity to claim their preferences, explain their contexts, and perhaps tell us something that we still don’t know.

### Did you face challenges collaborating between teams in different countries?

**NKC:** Given that the central phase of our research transpired amid the COVID-19 pandemic, we were compelled to predominantly work remotely. On one hand, this slightly impeded our progress, yet on the other, it fostered a shared experience for everyone, regardless of their geographical location. Ultimately, it was through a multitude of Zoom meetings, continuous monitoring, feedback loops, and daily conversations spanning nearly six months that we managed to gather and analyze our sample.

**CTG:** After the pandemic, this reality is normal. Now, Nicholas lives in Germany and me in Brazil and we talk every day. Our biggest problem is the time zone, but nothing too worrying. Our team has people from many cities, and we work together.

### How important was the collaboration to the success of the paper? How important do you think collaboration is in general to research?

**NKC:** None of this would have been possible without the assistance of our entire team. The type of analysis we aimed to conduct did not allow for significant automation, necessitating manual reading and parsing of all documents—a task that only the collaborative power of teamwork could accomplish. Thankfully, anyone can now access this distilled repository of knowledge through our dashboards.

**CTG:** As Nicholas said, the importance of collaboration is that without our team, the research wouldn’t exist. So we are very grateful to every collaborator. So much, thank you! We did it!

### What’s next for you and your collaborators?

**NKC:** Presently, our objective is to provide the community with two new research outcomes. Firstly, we are conducting a similar analysis, but with a focus on risk assessment. Here, our emphasis shifts from normative documents to the ML models themselves. We aim to evaluate the risks associated with numerous recently published AI models and applications, thereby constructing a comprehensive “risk landscape” of the AI domain. Secondly, we are determined to expand and enhance our “Worldwide AI Ethics” dataset and visualization panels. Therefore, in the future, we intend to revisit the data collection process and create “Worldwide AI Ethics 2.0,” featuring a more substantial sample size.

**CTG:** Now all we have to do is rest. Just kidding! We are working on methods and tools to help developers and enterprises build responsible artificial intelligence. And we are continuing our mission to alert the academic community and educate the general public about the risks of artificial intelligence.
